# C-reactive protein point-of-care testing in primary care—broader implementation needed to combat antimicrobial resistance

**DOI:** 10.3389/fpubh.2024.1397096

**Published:** 2024-07-19

**Authors:** Carl Llor, Andreas Plate, Lars Bjerrum, Ivan Gentile, Hasse Melbye, Annamaria Staiano, Oliver van Hecke, Jan Y. Verbakel, Rogier Hopstaken

**Affiliations:** ^1^Department of Public Health and Primary Care, University of Southern Denmark, Odense, Denmark; ^2^Via Roma Health Center, Catalonian Institute of Health, Barcelona, Spain; ^3^Institute of Primary Care, University and University Hospital Zurich, Zurich, Switzerland; ^4^Center for General Practice, Department of Public Health, University of Copenhagen, Copenhagen, Denmark; ^5^Department of Clinical Medicine and Surgery, Section of Infectious Diseases, University of Naples Federico II, Naples, Italy; ^6^General Practice Research Unit, Department of Community Medicine, The Arctic University of Norway, Tromso, Norway; ^7^Department of Translational Medical Sciences, University of Naples “Federico II”, Naples, Italy; ^8^Department of Public Health and Primary Care, Ghent University, Ghent, Belgium; ^9^NIHR Community Healthcare Medtech and IVD Cooperative, Nuffield Department of Primary Care Health Sciences, University of Oxford, Oxford, United Kingdom; ^10^LUHTAR, Department of Public Health and Primary Care, Academisch Centrum voor Huisartsgeneeskunde, Leuven & NIHR Community Healthcare Medtech and IVD Cooperative, Leuven, Belgium; ^11^GP Practice De Kuil, Hapert, Netherlands; ^12^Department of General Practice, CAPHRI School for Public Health and Primary Care, Maastricht University Medical Center, Maastricht, Netherlands

**Keywords:** C-reactive protein, anti-bacterial agents, inappropriate prescribing, drug resistance microbial, point-of-care testing, respiratory tract infections, complementary strategies, implementation

## Abstract

This study presents the perspective of an international group of experts, providing an overview of existing models and policies and guidance to facilitate a proper and sustainable implementation of C-reactive protein point-of-care testing (CRP POCT) to support antibiotic prescribing decisions for respiratory tract infections (RTIs) with the aim to tackle antimicrobial resistance (AMR). AMR threatens to render life-saving antibiotics ineffective and is already costing millions of lives and billions of Euros worldwide. AMR is strongly correlated with the volume of antibiotics used. Most antibiotics are prescribed in primary care, mostly for RTIs, and are often unnecessary. CRP POCT is an available tool and has been proven to safely and cost-effectively reduce antibiotic prescribing for RTIs in primary care. Though established in a few European countries during several years, it has still not been implemented in many European countries. Due to the complexity of inappropriate antibiotic prescribing behavior, a multifaceted approach is necessary to enable sustainable change. The effect is maximized with clear guidance, advanced communication training for primary care physicians, and delayed antibiotic prescribing strategies. CRP POCT should be included in professional guidelines and implemented together with complementary strategies. Adequate reimbursement needs to be provided, and high-quality, and primary care-friendly POCT organization and performance must be enabled. Data gathering, sharing, and discussion as incentivization for proper behaviors should be enabled. Public awareness should be increased, and healthcare professionals’ awareness and understanding should be ensured. Impactful use is achieved when all stakeholders join forces to facilitate proper implementation.

## 1 Introduction

### 1.1 Antimicrobial resistance

Antimicrobial resistance (AMR) is identified by the European Commission together with the Member States, as one of the top three priority health threats in the EU (1). Due to AMR, a growing number of infections that used to be effectively treated with antibiotics are becoming harder to treat as antibiotics become less effective (1). It was estimated that more than 35.000 people in the European Union (2) and more than 1.27 million people globally (3) died in 2020 and 2019, respectively, as a direct consequence of an infection due to bacteria resistant to antibiotics. The health impact of AMR in the European Union is comparable to that of influenza, tuberculosis, and HIV/AIDS combined (4). Currently, antibiotic resistance leads to high costs in the European Union, including € 1.5 billion annually for the healthcare systems (1). However, the costs of AMR will be borne most severely by future generations, impacting economic stability and increasing severe poverty as early as 2030 (5, 6) and causing an expected 10 million deaths per year due to drug-resistant infections by 2050 (7, 8).

Antimicrobial resistance is mainly caused by the inappropriate use of antibiotics. The majority of antibiotics are prescribed in the community. For many years, ecological studies have shown a link between the use of antimicrobials and the frequency of AMR. In 2021, the European Union population-weighted mean consumption of antibacterials for systemic use was 15.0 defined daily doses (DDD) per 1,000 inhabitants per day in the community and 1.4 DDD per 1,000 inhabitants per day in the hospital sector (9). The majority of antibiotics in primary care are prescribed for respiratory tract infections (RTIs), urinary tract infections, and skin infections, with RTIs accounting for more than half of these prescriptions. In addition, in many regions around the world, antibiotics are accessible without prescriptions. Despite being more prevalent in low-income countries, even in some high- and middle-income countries, non-prescription antibiotic provision is a concern (10). A large part of the antibiotics consumed in the community is estimated to be used inappropriately as the majority are self-limiting (viral or bacterial) infections (11, 12). Hence, antibiotic stewardship, which implies a more rational prescription and use of antibiotics, is crucial. A recent point-prevalence audit survey about doctors’ management of nearly 5,000 patients presenting with symptoms of an RTI in 18 European countries found that antibiotic prescribing rates varied considerably. In four countries, less than 20% of consultations led to antibiotic prescribing, and in six countries, more than 40% of consultations resulted in antibiotic prescribing (13). An Italian study including nearly 2000 patients with acute RTIs found antibiotic prescriptions were given in 67.3% of the consultations. Two-third of prescriptions were not according to the guidelines (14). Similarly, substantial over-prescribing has been found in United Kingdom primary care: in 41% of all acute cough consultations when experts advocated for it in only 10% and in 82% for bronchitis vs. the ideal of 13% (15). In primary care in Ireland, approximately 60% of patients with RTIs get antibiotics (16).

Antimicrobial stewardship is urgently needed to safeguard antibiotic effectiveness for future generations. Healthcare regulators and payers play a key role in enabling behaviors that ensure antibiotics are used effectively and sparingly (11, 17–20). C-reactive protein (CRP) point-of-care testing (POCT) is a simple, validated, and cost-effective tool supporting antibiotic prescribing decisions. When CRP POCT is combined with clear guidance, communication skills training, delayed prescribing, and other safety netting procedures, primary care management of RTIs will be improved. CRP can be used to assess the severity of inflammation and predict whether an infection is likely to be self-limiting or severe. Self-limiting infections, whether viral or bacterial, usually resolve on their own without further treatment. Interpreted together with signs and symptoms in the majority of patients with RTIs, the need for antibiotics can be ruled out. Guidance on the use of CRP POCT and complementary strategies to improve antibiotic prescribing has been recently published (21–23) (Table 1).

**Table 1:** Interpretation of C-reactive protein concentrations in adults with lower respiratory tract infection.

CRP values	Percentage of patients	Observations	Decision to treat or not with antibiotics
< 20 mg/L	70–75%	Self-limiting infection.	Do not prescribe antibiotics.
20–40 mg/L	10–13%	Most patients have a self-limiting infection. Evaluate specific patient risk.	Do not prescribe antibiotics for low-risk patients.
			Consider delayed antibiotic prescription in some cases.
41–100 mg/L	10–13%	Clinical picture most deciding. Evaluate specific patient risk	Consider antibiotic prescribing for patients with comorbidities that increase risk of complications (COPD, diabetes, vulnerable older people, etc.).
			Consider delayed antibiotic prescribing or re-consultation for patients without significant comorbidities.
> 100 mg/L	3–5%	Severe infection	Start antibiotic prescribing and consider hospital referral.

Adapted from Van Hecke et al. (21).

### 1.2 An evaluation of current evidence

#### 1.2.1 CRP POCT has been proven to safely reduce antibiotic prescribing for adult patients presenting symptoms of lower RTIs in addition to clinical assessment and is maximally effective when implemented together with complementary strategies

The effectiveness of quantitative CRP POCT to support the antibiotic prescribing decision has been extensively evaluated in the primary care setting for adult patients with lower RTIs. Several systematic reviews and meta-analyses provide an overview of available studies, concluding that CRP POCT significantly reduces the prescribing of antibiotics in adult patients with lower RTIs without compromising patient safety or satisfaction (18, 24–26).

- The latest Cochrane Review of Smedemark et al. in 2022 observed a mean reduction of antibiotic prescribing of 23% (RR 0.77; 95% CI 0.69–0.86) in adults covering 12 randomized trials (18).
- A European Network for Health Technology Assessment report found a reduction rate of antibiotic prescribing of 24% (RR 0.76; 95% CI 0.67–0.86) for randomized studies and 39% (RR 0.61; 95% CI 0.54–0.69) for observational studies (26).
- A reduction of antibiotic prescribing is safely achieved also in subgroups as in patients with acute exacerbations of chronic obstructive pulmonary disease (COPD) (27) (26% relative reduction, 20% absolute reduction, 57.0 vs. 77.4%) and in vulnerable, older adults in nursing homes (35% relative reduction, 29% absolute reduction, 53.5 vs. 82.3%) (28).

Further evidence underlines the importance of implementing CRP POCT together with complementary strategies and guidance such as safety netting advice on the interpretation of CRP values:

- Provide clear CRP value cutoff guidance: in randomized studies, an antibiotic prescription reduction rate of 32% (RR 0.68; 95% CI 0.63–0.74) was calculated in adults and 44% (0.56; 95% CI 0.33–0.95) in children once cutoff guidance was applied (24, 25).
- Offer communication skills training to physicians: while the use of CRP POCT has been shown to reduce antibiotic prescribing by approximately 42% in primary care (relative reduction; 22% absolute reduction, 31 vs. 53%) (29), additional studies have demonstrated that combining CRP POCT with communication skills training can increase the reduction of antibiotics prescribing to more than 60% (absolute reduction 44, 23 vs. 67%; RR 0.38; 95% CI 0.36–0.55) (29, 30). Recommended focus points for communication skills training are patient-centric consultation, sharing realistic expectations on disease and illness aspects, and shared decision-making (physician-patient) techniques.
- Enable the application of delayed prescribing techniques: this approach means that the patient receives a prescription with the instruction to “fulfill at their discretion” based on their monitoring of symptoms during a certain period. According to the 2023 Cochrane review of Spurling et al., delayed prescribing resulted in reduced antibiotic use compared to cases of immediate antibiotic prescription (30 vs. 93%), with similar re-consultation rates (31). In a Dutch randomized study, delayed prescription combined with CRP POCT resulted in a significant absolute reduction of the fill rate of 49% compared to delayed prescription without CRP POCT (32).
- Encourage the use of decision aids: research has shown that using decision aids (a pamphlet with a diagram or flow chart) does not take much additional consultation time (on average 1.5 min) (19), does not negatively impact patient satisfaction or health outcomes, and has the potential to reduce antibiotic prescription by 9.1% (33). Increasing understanding of and engagement with the treatment decision could positively influence compliance, which is especially important in areas with easy access to antibiotics.

#### 1.2.2 Evaluation of evidence supporting CRP POCT effectiveness in safely reducing antibiotic prescribing for children presenting with an acute RTI

Evidence regarding the effectiveness of CRP POCT in safely reducing antibiotic prescribing for children presenting symptoms of RTIs in primary care is still limited. Recent systematic reviews indicate a clear potential to safely reduce antibiotic prescribing for children with acute RTIs:

- As mentioned before, Verbakel et al. and Martínez-González et al. calculated an antibiotic prescribing reduction rate of 44% for children in randomized studies once CRP cutoff guidance was applied, and this without any negative effect on patient outcomes or healthcare processes (RR 0.56, 95% CI 0.33–0.95) (24, 25).
- In 2020, Van Hecke et al. concluded that there is emerging evidence that CRP POCT can be effective at reducing antibiotic prescribing for children with acute RTIs in low- and middle-income countries but that evidence of the effectiveness in high-income countries is not as abundant (34).
- In the updated systematic review on the impact of the use of CRP on antibiotic prescribing by Smedemark et al., a total of four randomized clinical trials including 2,335 children collectively found that CRP rapid testing reduces the number of children given an antibiotic prescription by 22%, with this effect being primarily seen in low- and middle-income countries (0.78, 95% CI 0.67–0.91) (18).

While CRP POCT could be a useful tool to reduce antibiotic prescribing for children with RTIs, especially when clear guidance is provided and effective communication strategies are used, it would be useful to generate more context-specific evidence to increase confidence for broader application, and understand best practices in pediatric ambulatory care settings worldwide, and provide clear, evidence-based cutoff guidance to assist the antibiotic prescribing decision.

### 1.3 Healthcare economics of CRP POCT

Antibiotic overuse in humans and AMR are strongly correlated (35–37), and the total global economic direct and indirect costs of AMR due to resistance in five pathogens are already very high, estimated at USD 2.9 billion in the United States alone (38). According to the OECD, the cost of treating complications due to AMR can exceed USD 28.9 billion every year adjusting for purchasing power parity across 34 OECD and EU/EEA countries (39). This is calculated for a period from 2021 up to 2050. Most of the costs are caused by longer hospitalizations. Thus, the costs caused by AMR are high. On the other hand, every USD 1 invested in a mixed policy package across the health and food sectors generates returns equivalent to USD 5 in economic benefits achieved through reductions in health expenditure and increased productivity at work (39).

Unfortunately, the costs associated with AMR are rarely included in economic evaluations of antimicrobials and interventions such as diagnostics and vaccines that affect their consumption (38, 40). The cumulated economic costs of a single course of antibiotic treatment including the costs of AMR, however, are difficult to quantify precisely, and relatively few attempts to do so have been made (41, 42). Cumulative costs will vary depending on the specific context. Models for calculation are available, for example, Shrestha et al. calculated that a standard course of broad-spectrum penicillin has a cumulative economic cost of USD 9.30 in the United States and USD 10.40 in Thailand (38).

Reduced antibiotic prescribing has ecological benefits and leads to reduced selection of resistant strains. Furthermore, healthcare economics have demonstrated that implementing CRP POCT for RTIs is cost-effective:

- Oppong et al. (41) calculated a 70% probability that CRP POCT would be cost-effective in the primary care setting in Norway and Sweden.
- Holmes et al. (43) calculated an 84% probability that CRP POCT would be cost-effective in primary care in the United Kingdom for patients presenting symptoms of lower RTIs and that adhering to clinical guidance increased cost-effectiveness.
- Hunter concluded that cost savings and quality-adjusted life year improvements outweighed the incremental costs of performing CRP POCT in the primary care setting in England (44).
- Fawsitt et al. (16) concluded that in primary care in Ireland, CRP POCT (with and without communication training) was more costly on a 5-year horizon, but also more effective, with potential long-term budget savings depending on the implementation scenario.
- The OECD calculated the *per capita* cost for the implementation of CRP POCT of USD 0.53–2.15 across countries. Those costs cover the cost of buying the CRP POCT devices, costs related to training the prescribers on the clinical guidelines related to the use of CRP tests and informational materials, as well as some administrative expenses and expenses covering monitoring and evaluation activities at the national and local levels. Additional costs to account for any additional time that prescribers may spend to perform the CRP POCT have not been included in these estimates (39).

Van der Pol et al. developed a model for general practice practices in the Netherlands comparing the use of a hypothetical diagnostic strategy to continuing the current standard of care for all patients with suspected acute RTIs. They concluded that primary care costs on consultations will be raised by 9 and 19% when consultation is priced 5 and 10€, respectively, while antibiotic consumption and AMR will be lowered (45). The price per consultation includes the costs of a test analyzer itself and materials used for the test and also costs related to the depreciation and quality assurance related to the use of the hypothetical diagnostic strategy. The efficiency of the CRP POCT device will be higher than accounted for in those studies since many CRP devices have the option to test more parameters for different illnesses on the same device.

Wubishet et al. (20) included 12 studies in their systematic review of economic evaluations of interventions aimed at reducing inappropriate antimicrobial prescribing in primary care. They concluded that there were significant variations in the cost-effectiveness of antimicrobial stewardship interventions across studies and depending on the inclusion of cost components such as the cost of AMR. However, communication skills training and CRP POCT were frequently found to be cost-effective or cost-beneficial for reducing inappropriate antimicrobial prescribing (20).

## 2 Policy options and implications

### 2.1 Implementation models

An effective implementation strategy needs to be chosen depending on the specific context within a country or region. There are a few operating models for CRP POCT implementations that have been successful. However, the strategies may differ from country to country, and there may be more feasible options.

1. A physician acquires a CRP POCT device (independently or through local health organizations) and manages the installation, information system integration, and initial training, potentially with support from the device supplier. Once operational, physicians are responsible for managing the supply of consumables and ensuring quality themselves. The physician may be supported by a medical assistant, nurse, or other in performing the tests and managing supply and quality.
2. A laboratory or diagnostic service organization (“lab”) acquires the CRP POCT device and makes it available for the physician (and, if relevant, supporting staff) to use. The laboratory supports supply management of consumables, quality assurance, device maintenance, linking of information systems, and practical instruction, training, and certification of the users.
3. A scientific national institute or non-profit organization provides quality improvement services for POCT such as guidance and education through site visits, telephone consultations and courses, advice about what instruments to buy, and external quality assessment programs.

Depending on the chosen model, various practical aspects need to be in place to ensure a successful and sustainable implementation:

- Implementation should be ratified, facilitated, and/or supported by policymakers by providing clear frameworks. If laboratories are involved, contracts/service agreements will be needed between the lab and the diagnostic company (clearly outlining roles, responsibilities, and requirements), as well as between the physicians, the device supplier, and other involved parties. These frameworks will enable collaboration, accelerate implementation, and contribute to the quality of testing and training.
- If laboratories are involved, IT integration requirements and related costs for inter-system communication and quality control (i.e., middleware set-up, system linkage) are to be considered for the connection of the CRP POCT device between physician practice and lab to input test results into a shared electronic information system (46).
- Proper training of staff, both on the use of the device and the interpretation of results, is key for successful implementation.
- Quality assurance, including device verification and maintenance as well as the monitoring of correct use, is key and may be difficult for physicians/practices to manage independently. Support could come from a number of third parties such as labs, scientific national institutes (i.e., Sciensano in Belgium, RILIBÄK in Germany, and NOKLUS in Norway), medical devices companies, governmental authorities, or others.

Several Nordic countries, Switzerland, and the Netherlands are examples where CRP POCT has been successfully implemented in primary care.

In Norway, the use of CRP POCT started with the launch of a semi-quantitative test, Nycocard^TM^ CRP Visual, in 1989 (47) followed in 2000 by the launch of the Nycocard^TM^ CRP Single Test for the NycoCard Reader giving quantitative results (48). Around 1992, the costs attached to CRP POCT became reimbursed. Approximately 60% of all RTI-related consultations at primary care involve a CRP POCT, and in out-of-hour services, CRP POCT is performed in 70% of RTI-related consultations (49). A national guideline “antibiotics in primary care” gives instructions about the use of CRP and the interpretation of values when antibiotic prescribing should be considered for suspected lower RTI (50). Since 2012 there has been a marked steady decline in total antibiotic use with a reduction of 33% (51) and resistance rates are stable and low.

In Sweden, CRP POCT has also been used for 30 years in managing patients with RTIs (52). It is widely used in nearly two-thirds of RTI consultations (53). National guidelines advocate the use of the CRP POCT in primary care RTI consultations primarily for unclear lower RTIs (54, 55). In Sweden, primary care is often provided in group practices, called primary healthcare centers, with an average of four general practitioners (GPs) (56, 57). The primary healthcare centers, not the GP, are charged for the costs (58) when using diagnostic tests. The Swedish Strategic Program Against Antibiotic Resistance (STRAMA) illuminated the problem of resistant bacteria and increased awareness of antibiotic resistance (59). Levels of antibiotic use and resistance in Sweden are among the lowest among the European Union (EU) countries. Between 1992 and 2016, the number of antibiotic prescriptions in outpatient care, including primary healthcare, decreased by 43% (59).

In Denmark, CRP POCT for RTIs has been implemented for more than 20 years, and GPs have been paid a fee for performing a CRP test (60, 61). The first step was the implementation of CRP POCT in the national guidelines of the Danish College of General Practitioners (62). Danish Regions facilitated quality improvement programs based on audits and feedback on diagnostics and antibiotic use. The quality intervention program was organized by Audit Project Odense (APO). A nationwide retrospective cross-sectional register-based study found that between 2015 and 2017, approximately half of the antibiotic prescriptions for adults in general practice (49.6%) had an RTI stated as the indication, and a CRP test was performed in 45.2% of these cases (61). A 25% reduction in the consumption of antibiotics in primary care during 2011–2020 has been observed (63).

In Switzerland, CRP POCT has also been widely used for many years (64). Already in 2006, CRP has been the most used test for acute RTIs. According to Briel et al. for 42% of patients with acute RTI, at least one test was carried out by GPs in 2004, and in 35%, it was a CRP test (65). In 2022, a survey among GPs found that most of them (92–98%) selected a CRP POC test alone or combined with other diagnostics in the management of RTI (64). For participation in proficiency testing, practices hold a “practice laboratory” certificate. The CRP POCT is reimbursed and used in adults and children with acute infections (64). Switzerland has one of the countries lowest prescribing rates in Europe (66).

In the Netherlands, guidelines from the Dutch College of GPs recommend CRP POCT for the management of adults with acute cough (67, 68), suspected diverticulitis (69), and exacerbations of COPD (70) since 2011. Healthcare solutions recommended in clinical guidelines are considered as usual care and are almost automatically included in the “Basic Package of Care” and thus will get reimbursed. A large majority of the Dutch GPs have contracted a (regional) hospital or primary care lab for collaboration on POCT (CRP and possibly other tests). GPs are outsourced by the lab on purchase, organizational aspects, and logistics and can fully concentrate on patient care without financial incentives. In return, the lab can reimburse the test costs at the insurance company. The GPs who do not collaborate with a laboratory (a minority) have purchased the device themselves and can ask the insurance company for reimbursement of the actual test costs per patient. Since 2011, almost all Dutch GPs have used CRP POCT routinely 24/7 with high satisfaction of users and patients (71). The already low rate of antibiotic prescribing further decreased by 14%. Causality can only be suspected, but the use of CRP POCT has probably contributed to this decrease (72).

The implementation of POCT involves several actors and processes. Dewez et al. (72) evaluated which factors contribute to high vs. low adoption of CRP POCT in the Netherlands and England. They used a comparative qualitative case study approach and collected data through a review of documents and interviews with stakeholders. In both countries, early adopters of the tests advocated for its implementation by generating robust evidence and engaging with all relevant stakeholders. This led to the inclusion of CRP POCT in clinical guidelines in both countries. However, only in the Netherlands did this result in the reimbursement of the test costs. In addition, the better integration of health services enabled operational support from laboratories to general practice practices in the Netherlands. In contrast, in England, the development of a reimbursement program was prevented by the funding constraints of the National Health Service and the prioritization of alternative and less expensive antimicrobial stewardship interventions such as practice visits, needs assessment, peer feedback, and audits (72, 73). In addition, the lack of integration between health services limits the operational support to general practice practices.

Lingervelder et al. (74) compared the implementation of POCT in The Netherlands and Norway, where POCT is more widely implemented, with that in England and Australia, where POCT is not widely implemented. They analyzed the structure of healthcare operations and the transactions between stakeholders and found that the biggest challenge for countries with low POCT uptake was the lack of effective communication between the several organizations involved with POCT as well as the high workload for GPs aiming to implement POCT. They concluded that setting up a single national authority may be an effective step toward realizing the full benefits of POCT.

### 2.2 Current policies

In June 2023, the Council of the European Union (75) and the European Parliament (76) published recommendations on EU actions to combat AMR. They encourage their member states to support the prudent use of antimicrobial agents, in healthcare settings, including primary healthcare settings and long-term care facilities, and community care in particular. One recommendation is the improvement of the availability, cost-effectiveness, and timeliness of diagnostic tests (76). Specific consideration is given to rapid tests conducted prior to the prescription of antimicrobial treatment, in particular in primary care, to ensure the optimal prescription. Where possible, antibiotic prescription should be restricted to face-to-face consultations. The European Parliament calls on the Commission “to work toward the development of EU guidelines including recommendations to strive to always carry out diagnostic tests, including rapid tests when available, prior to the prescription of antimicrobial treatment, … to ensure their optimal and most prudent use” (76).

C-reactive protein point-of-care testing is infrequently mentioned in national AMR (77–80) plans or by NGOs such as the WHO (81, 82) and the ECDC (83). Rather tests to identify the cause of an infection or susceptibility testing are usually recommended. Several policies promote at least the use of rapid diagnostic tests, for example, the French national AMR plan (79). The German government promotes in DART 2030 (78) the use of rapid diagnostic tests to differentiate between viral and bacterial infections as the initial investigation at the point of care.

An exception is the report from the OECD. The report “Stemming the Superbug Tide: Just A Few Dollars More” (6) gives detailed insight into the evidence and implementation of CRP POCT. The new OECD report “Embracing a One Health Framework to Fight Antimicrobial Resistance” (39) calls for scaling up the use of rapid CRP POCT in addition to other community-based interventions such as delayed antimicrobial prescriptions, introducing financial incentives to optimize antimicrobial use, scaling up mass media campaigns, and scaling up prescriber training. The availability of CRP POCT should be increased in ambulatory care settings in combination with antibiotic treatment guidelines. The modeled increase in the availability of CRP is assumed to reduce immediate antibiotic prescribing by 32% in adults and 46% in children under 18 years of age, and the intervention is assumed to yield immediate effects on antibiotic prescribing behaviors.

The WHO Policy Brief 63 is another exception. It highlights CRP POCT as an example of rapid laboratory testing, which should be made available to guide antibiotic prescription within a few minutes (84).

An overview of available CRP POCT, their use in primary care, and the reimbursement in specific countries can be found in addition to other important information in the review of the European Network for Health Technology Assessment (2019) (26) and more recently in the report of the PHG Foundation’s independent research and analysis for Health Action International (2023) (85).

## 3 Recommendations

Globally, societies must strive toward better stewardship of antibiotics. This, however, requires significant shifts in thinking relevant to the primary care setting. Re-evaluation is needed by many stakeholders including physicians, patients, payers, and policymakers to safeguard the effectiveness of antibiotics (86). Antibiotic stewardship can most efficiently be achieved by acting on several fronts and deploying several complementary strategies in parallel (87).

Table 2 covers the recommendations of the authors for stakeholders who can turn the tide. They largely correspond to the Antimicrobial Stewardship interventions suggested by the WHO (81) and a group of experts from Australia (88).

**Table 2:** Suggested antimicrobial stewardship interventions.

Include CRP POCT in Guidelines and AMR reports and Action Plans. Ensure CRP POCT is implemented together with complementary strategies. Provide adequate reimbursement for CRP POCT and complementary strategies. Enable high-quality and primary care friendly POCT organization and performance. Enable data gathering, sharing, and discussion as incentivization for proper behaviors. Generate context-specific pilots and evidence where needed. Increase public awareness. Ensure healthcare professionals’ awareness and understanding.

### 3.1 Include CRP POCT in guidelines, AMR reports, and action plans

Having access to a CRP POCT, on its own, is not enough to reduce antibiotic prescribing. Clear guidance to support physicians in their decision-making is needed to achieve safe and meaningful reductions. Several national respiratory guidelines (50, 54, 55, 62, 68, 89–98) from Belgium, Denmark, Germany, Norway, the Netherlands, Spain, Sweden, Switzerland, the United Kingdom, and South Africa have already implemented CRP POCT. Most of them provide cutoff recommendations, usually 20, 40, and/or 100 mg/L (Table 1). A few do not recommend any cutoffs (55, 94, 95). A clear cutoff guidance is essential as outlined before and should include detailed guidance on the interpretation of CRP values and guidance for the selection of appropriate patients, who would benefit from testing as recently provided by the authors. An update of national guidelines is recommended once CRP POCT for the guidance of antibiotic prescribing for RTIs is not yet or insufficiently implemented.

### 3.2 Ensure CRP POCT is implemented together with complementary strategies

To maximize the safe reduction of antibiotic over-consumption, CRP POCT should be implemented in conjunction with several complementary strategies (31–33, 99, 100). As discussed previously (21–23), maximal impact can be gained when policymakers offer communication skills training to medical students and physicians, enable the application of delayed prescribing techniques, and encourage the use of decision aids during the consultation.

### 3.3 Provide adequate reimbursement for CRP POCT and complementary strategies

Lack of reimbursement and costs associated with CRP POCT for physicians have been identified as the main reason for non-adherence to guidelines that recommend the use thereof (11, 101). In general, a neutral reimbursement level that balances costs for physicians, health insurance, and the healthcare system is recommended to ensure the proper use of CRP POCT. Neutral reimbursement levels would not incentivize overuse, nor de-incentivize use of the tests when they could be beneficial, thus maximizing the systemic effectiveness of an implementation. Slight over-reimbursement might be a solution in the short term if the healthcare-economics business case permits it, to encourage the use of CRP POCT, especially in an early adoption stage.

Table 3 covers the elements of cost to consider when determining appropriate reimbursement levels. Actual costs will differ per country/region. Adequate reimbursement should go to the party that is performing the action or service and thus carrying the cost, so in the case of collaborations with labs or other third parties, reimbursement should be split.

**Table 3:** Costs to consider when determining appropriate reimbursement levels.

Purchase of the device and depreciation. Costs of consumables. User time to perform CRP POCT and extra time for consultation and dedicated space for CRP POCT device and consumables. Training of CRP POCT users. Inventory, logistics, and stock management of consumables costs. Quality assurance and maintenance of CRP POCT device, that is, internal and external proficiency testing according to instructions in the package insert and local rules. If a lab is involved, support in person or via call line, for example, for travel to sites, training, quality assurance, and troubleshooting. IT infrastructure, that is, connecting data between GP practice, patient, and lab for continuous validation of results and integration of information within a single system.

### 3.4 Enable high-quality and primary care-friendly POCT organization and performance

Though POCT devices are usually designed to be easy to handle also by non-lab-staff, the implementation of POCT may be facilitated for physicians/practices if they are accompanied by specialists from laboratories, national institutes, or non-profit organizations. Those specialists may ensure that the devices are used as intended and according to the local standards. They may support with consultation, giving advice regarding where to place the device best, for example, not next to a heater, or about the cooling logistics for the tests, perform training, and offer troubleshooting. Especially they may keep control of the quality assessment to ensure the establishment of quality standards.

### 3.5 Enable data gathering, sharing, and discussion as incentivization for proper behaviors

Enable or facilitate the gathering and sharing of data and encourage ongoing peer-to-peer interactions/discussions, small group meetings or focus group discussions (102–104). The data should include comparative numbers between practices from within a region, normalized per patient type. These could compare relative antibiotic prescription behavior, the number of CRP POCTs performed, and the satisfaction of patients, physicians, and supporting staff. Data should be tracked over time, including baseline numbers from before the implementation of CRP POCT and complementary strategies.

Additional positive reinforcement through recognition of best performers may be considered in countries where CRP POCT is not yet established or in the early stages of adoption, and antibiotic prescribing rates are high. This can be an effective way to influence physicians’ behaviors according to the nudge theory, a concept that proposes positive reinforcement and indirect suggestions as ways to influence the behavior and decision-making of groups or individuals (105, 106).

Financial incentives are not recommended, since good performance indicators for this subject are notoriously difficult to define, track, and interpret, and poorly defined and implemented incentivization strategies can encourage wrong behaviors and be counterproductive.

Recently, the Joint Programming Initiative on Antimicrobial Resistance—Primary Care Antibiotic Audit and Feedback Network (JPIAMR-PAAN) developed best practice guidelines for peer comparison audit and feedback for antibiotic prescribing in primary care in high-income countries (107). They defined four categories with 13 best practice recommendations. For example,

- physicians should feel supported rather than threatened;
- potential barriers to success as local data availability and validity and other situational factors should be considered;
- all primary care prescribers should be included in utilizing an opt-out approach to the delivery of feedback reports;
- behavior change messaging should be included alongside peer comparison;
- antibiotic audit and feedback reports in primary care should be repeated with updated data over time using multiple forms of communication;
- individual feedback should be delivered confidentially to prescribers and in general, be delivered by a respected authority figure or colleague; and
- feedback indicators for antibiotic prescribing in primary care should target reductions in antibiotic initiations, prolonged antibiotic duration, and/or unnecessary broad-spectrum antibiotics.

As highlighted by the JPIAMR-PAAN, inappropriate antibiotic prescribing behavior is complex (107). On the one hand, there are clinically important factors, such as AMR and the risk of adverse drug reactions. On the other hand, there are emotional factors driving unnecessary prescribing, such as perceived patient expectations, time constraints, habits, and fear of patients worsening. Thus, multifaceted approaches to antibiotic prescribing in primary care to facilitate a change in prescribing are essential. Audit and feedback should be implemented alongside clinical decision support, point-of-care diagnostics, patient and prescriber education, and safety netting procedures.

### 3.6 Generate context-specific pilots and evidence where needed

Despite the existing evidence and seeming consensus regarding the usefulness of CRP POCT and other strategies in reducing antibiotic over-prescription in primary care settings, implementation and adoption are still limited. This is linked, in part, to the lack of context-specific evidence for the effectiveness of CRP POCT use. Countries/regions may need to set up pilot projects or clinical research projects to generate a local, context-specific assessment of efficacy and cost-effectiveness and understand barriers and facilitators to scaling-up.

### 3.7 Increase public awareness

Communication with the broader public—the patients and parents—will be key (7). In many countries, it may be necessary to build awareness and understanding that the inappropriate use of antibiotics is the main cause of AMR to enable and reinforce the adoption of desired behaviors. It is important that patients and parents understand their key role in reducing antibiotic consumption and that antibiotics are not antipyretics.

A multi-channel communication approach is suggested, deploying messages simultaneously via various target audiences such as parents, adults, and teenagers. Campaigns should be repeated every 3–4 months for continuity and higher impact, and strongest during (possible) periods with the highest prevalence of RTIs.

### 3.8 Ensure healthcare professionals’ awareness and understanding

Healthcare professionals will play a key role in both reducing the number of antibiotic prescriptions and also in dialoguing with patients and parents, spreading the messages related to antibiotic stewardship. Physicians need to understand the basics of AMR, the risks related to the misuse of antibiotics and what constitutes appropriate use, and the clinical benefits of CRP POCT in reducing diagnostic uncertainty and the antibiotic prescription decision-making process.

Physicians need to get the chance to learn of, and experience, the support of CRP POCT in daily practice. Although AMR will be seen as a problem, on a day-to-day basis this is mostly not experienced in the consultation room with a patient with a lower RTI. Instead, diagnostic uncertainty, time pressure, and perceived antibiotic beliefs of patients are important concerns that are nowadays still mostly silenced by prescribing an antibiotic. CRP POCT as a tool and a low CRP test result at hand will encourage both the patient and the physician to change this (high) antibiotic prescribing behavior.

Adapted communication and training programs (19, 26) should be utilized for pilot projects and during the broader roll-out of the CRP POCT to primary care to ensure the tests are deployed effectively and in combination with the strategies that maximize impact. Continued communication is recommended to keep interest in CRP POCT high and to continue convincing physicians of its benefits and increase use.

## 4 Conclusion

Antibiotic over-prescribing in primary care is significant and heavily contributes to the rising AMR worldwide. Most unneeded antibiotics are prescribed for RTIs. CRP POCT in primary care, added after the clinical examination, is proven to be effective in safely reducing the use of antibiotics. It can be currently considered the best available option to combat AMR in primary care. It makes healthcare economically efficient and is most effective when implemented in combination with clear guidance, advanced communication training, and delayed prescribing.

Healthcare regulators and policymakers are encouraged to enable the broader application of CRP POCT and complementary strategies in primary care for patients with RTIs, by updating and implementing national guidelines, and AMR action plans, ensuring adequate reimbursement, and taking other reinforcing actions such as data sharing and broad communication and education into account.

## Author’s note

The authors represent the ENASPOC expert group: https://www.enaspoc.com/the-expert-group.

## Author contributions

CL: Writing – original draft. AP: Writing – original draft. LB: Writing – original draft. IG: Writing – original draft. HM: Writing – original draft. AS: Writing – original draft. OH: Writing – original draft. JV: Writing – original draft. RH: Writing – original draft.
